# Prevalence of depression and anxiety among general population in Pakistan during COVID-19 lockdown: An online-survey

**DOI:** 10.1007/s12144-022-02815-7

**Published:** 2022-02-08

**Authors:** Irfan Ullah, Sajjad Ali, Farzana Ashraf, Yasir Hakim, Iftikhar Ali, Arslan Rahat Ullah, Vijay Kumar Chattu, Amir H. Pakpour

**Affiliations:** 1 Kabir Medical College, Gandhara University, Peshawar, Pakistan; 2 Ziauddin Medical University, Karachi, Pakistan; 3 Department of Humanities, COMSATS University, Lahore, Pakistan; 4 Paraplegic Centre, Hayatabad, Peshawar, Pakistan; 5 Department of Medicine, Northwest general hospital and Research Centre, Peshawar, Pakistan; 6 Department of Medicine, Temerty Faculty of Medicine, University of Toronto, Toronto, ON M5S 1A8 Canada; 7 Center for Transdisciplinary Research, SIMATS, Saveetha University, Chennai, India; 8 Department of Community Medicine, Faculty of Medicine, Datta Meghe Institute of Medical Sciences, Wardha, India; 9 Department of Nursing, School of Health and Welfare, Jönköping University, Gjuterigatan 5, 553 18 Jönköping, Sweden

**Keywords:** Depression, Anxiety, COVID-19, General population, Cross-sectional design, Gender, Mental health

## Abstract

The present study's aim is to find the prevalence of two of the common indicators of mental health - depression and anxiety – and any correlation with socio-demographic indicators in the Pakistani population during the lockdown from 5 May to 25 July 2020. A cross-sectional survey was conducted using an online questionnaire sent to volunteer participants. A total of 1047 participants over 18 were recruited through convenience sampling. The survey targeted depression and anxiety levels, which were measured using a 14 item self-reporting Hospital Anxiety and Depression Scale (HADS). Out of the total sample population (*N*=354), 39.9% suffered from depression and 57.7% from anxiety. Binary logistical regressions indicated significant predictive associations of gender (*OR=1.410*), education (*OR=9.311*), residence (*OR=0.370*), household income (*OR=0.579*), previous psychiatric problems (*OR=1.671*), and previous psychiatric medication *(OR=2.641)*. These were the key factors e associated with a significant increase in depression. Increases in anxiety levels were significantly linked to gender (*OR=2.427*), residence (*OR=0.619*), previous psychiatric problems (*OR=1.166*), and previous psychiatric medication (*OR=7.330*). These results suggest depression and anxiety were prevalent among the Pakistani population during the lockdown. Along with other measures to contain the spread of COVID-19, citizens' mental health needs the Pakistani government's urgent attention as well as that of mental health experts. Further large-scale, such as healthcare practitioners, should be undertaken to identify other mental health indicators that need to be monitored.

## Introduction

In early December 2019, many cases of pneumonia caused by a novel beta coronavirus (the 2019 novel coronavirus) were identified in Wuhan, the capital city of Hubei, China (Guan et al., 2020). This virus has been named severe acute respiratory syndrome coronavirus2 (SARS-CoV-2), which displays phylogenetically identical characteristics to severe/acute respiratory syndrome coronavirus (SARS-Co-V) (Lu et al., 2020). The World Health Organization (WHO) declared COVID-19 a global pandemic on 11 March 2020, when the registered cases of COVID-19 reached 118,000, and the number of deaths reached 4291 in 114 countries (WHO, 2020). Amid epidemics, there is a growing sense of fear among individuals of becoming infected with viral diseases, which further causes anxiety and depression (Ahorsu et al., 2020; Hall et al., 2008). Anxiety is defined as the body's normal response to stress (Holland, 2018). Depression, on the other hand, is defined as a lack of interest in everyday tasks. It is hypothesized that persons who are exposed to a pandemic without immunization will experience anxiety, tension, and depression as a result of their fear of the unknown (in this case, the coronavirus) (Lin et al., 2021).

Since the outbreak of COVID-19, a large number of studies have been conducted on people’s mental health during lockdown and quarantine situations, particularly on ways to cope with the spread of these conditions. All the research has led to the conclusion that various restrictions to an individual’s behavior can have an adverse effect on their mental health. For example, a study conducted by Sprang and Silman (2013) showed that 25% of isolated parents and 30% of isolated children had symptoms of post-traumatic stress disorder (PTSD). A Korean by Jeong et al. in 2016 found that 7.6% of patients displayed symptoms of anxiety during the epidemic of Middle Eastern Respiratory Syndrome (MERS). In addition, similar results were reported in Canada during the 2003 severe acute respiratory syndrome (SARS) outbreak (Reynolds et al., 2008).

Fear, anxiety, desperation, and helplessness can be associated with epidemic outbreaks of infectious diseases, especially when infection and death rates are reported to be high (Ashraf et al., 2021; Rajabimajd et al., 2021). Tension spikes in the general population during infectious disease outbreaks, bringing not only poor mental health but also major economic consequences in the social and household sectors (Smith et al., 2019). The travel bans in China during the outbreak of SARS in 2003 and avian influenza in 2013 had a huge impact on the business sector and the jobs of individuals (Wishnick, 2010). The mental health of the general population can be affected by the COVID 19 pandemic and is of great importance from various aspects (Xiang et al., 2020). Due attention needs to be paid to the psychological traumas, and mental health problems experience in the general population. Controlling situations such as lockdowns can provoke anxiety responses and increase the fear and prejudice against infected and affected persons (Person et al., 2004). Studies examining the effect of COVID-19 on mental wellbeing not only highlight problematic areas but can also generate ways to provide health care services with the necessary information and support to deliver mental health treatment to those in need. Though a large number of studies have explored mental health in the particular context of mental health outcomes, yet there is a paucity of research assessing the prevalence of anxiety and depression symptoms relating to socio-demographic factors. Furthermore, the present study will be a valuable addition to the existing literature on the cultural aspect of mental health and depression.

## Methodology

### Study Design and Participants

A cross-sectional study was conducted from 5 May to 25 July 2020 in Pakistan, targeting the general population. Online survey was accompanied by a self-administered questionnaire. The online survey was distributed by commonly used social media such as Facebook, WhatsApp and Telegram. Participants were also asked to share the online survey with their peers to obtain a more normal distribution and representative sample. To control the possible confounding factors, certain inclusion criteria were devised. Participants had to be (i) Pakistani nationals residing in the country since the outbreak of the corona pandemic, (ii) at least 18 years of age, (iii) able to speak Urdu as their first language, and (iv) not previously diagnosed with a psychological or psychiatric disorder or on any medications for the same. The exclusion criteria include: (i) Non- Pakistani nationals inside the country as well as Pakistani national living abroad, (2) anyone less than 18 years of age, (iii) anyone with a prior diagnosis of depression or anxiety disorder or any other mental health issue (iv) anyone who is on anti-psychotic or psychiatric medications. Dropouts and participants who provided insufficient or incomplete data were excluded from the study. The final sample comprised 1047 participants recruited through convenience sampling. The study was approved by the ethics committee of COMSATS University Lahore (REF: CUI/LHR/HUM/178) and carried out in accordance with the human research ethics outlined in the Helsinki Declaration 1975. The online survey comprised three sections; informed consent, demographic information, and study tools. Informed consent was provided by all the participants before completing the online survey. The participants were assured that their participation in the study was voluntary and were free to withdraw from the survey at any point without any privacy concerns. The survey remained anonymous to assure the reliability, replicability and confidentiality of the data

### Measures

i. *Demographic Questionnaire:* With the help of a self-reporting standard questionnaire, socio-demographics parameters of the participants such as age, gender, marital status, education, region, area of residence, occupation, monthly household income, and smoker status were collected.
ii. *Hospital Anxiety and Depression Scale (HADS):* The HADS was used to assess anxiety and depression in the study sample (Waqas et al., 2019). HADS is a valid measure for assessing mental health outcomes in terms of depression and anxiety and is widely used locally and internationally. The scale comprises 14 items equally distributed to assess anxiety (e.g., *“I feel tense or wound up”*) and depression (e.g., “*I still enjoy the things I used to enjoy*”) through responses to statements. Two of the items are reverse coded (items 7 and 10) to cross-check the random responses. Each item is rated on a four-point Likert scale (0 to 3 with diverse descriptions for each item) with total scores ranging from 0 to 21. High scores are an indicator of a high level of depression and anxiety. Scores on HADS can be used on a continuum and as categorical as well (e.g., normal=0-7; mild=8-10; moderate=15-21 and severe=15-21). The present study showed a good fit for the alpha coefficient for total HADS (α=.85), depression (α=.72) as well as anxiety (α=.84) subscales.

### Data Analysis

The data were analyzed using IBM SPSS Statistics V.26.0. All the data were coded in SPSS, and invalid data (e.g., random responses, incomplete responses, and repetitive responses) were dealt with using missing values analysis and outliers’ analysis in SPSS. We ran descriptive statistics -means, standard deviation, percentages, and frequency distribution - to estimate the descriptive characteristics of the study variables. First, the association between independent and dependent variables was determined using the Chi-square test of association. In addition, we ran logistical regression analyses to evaluate the degree of association of socio-demographic characteristics with depression and/or anxiety. The level of significance had a p-value < 0.05 and a confidence interval (CI) of 95%.

## Results

Of the total 1047 participants, a majority 550 (52.5%) were females and 497 (47.5%) were males. The vast majority (85%) of them were aged 18 - 30 years and the remaining (15%) were over 30 years of age indicating that the majority of the sample comprised of young adult population. The mean age (S.D) was found to be 25.76 ± 11.262 years which also indicates the higher use of social media platforms by this age groups. The participants resided in all provinces: 53.7% lived in Sindh, 22.5% in Punjab, 12.9% in Khayber Pukhtoonkhawah, 10.4% in Islamabad, 0.3% in Azad Jammu Kashmir, 0.2% in Gilgit Baltistan, and 0.1% Balochistan. More than 1/3 of the study participants (77.8%) were unmarried. Only 221(21.1%) were married, 6 (0.5%) were separated/divorced and 5 (0.4%) widowed (see Table 1).

**Table 1:** Demographic Characteristics of participant

Characteristics	*f*	%	Characteristics	*f*	%
**Age (years)**			**Smoker**		
18-30	890	85	Non smoker	918	87.7
> 30	157	15	Current smoker	92	8.8
**Gender**			Former smoker	37	3.5
Male	497	47.5	**Household monthly income**		
Female	550	52.5	500000 ≥	342	32.7
**Marital status**			500001-100000	305	29.1
Single	815	77.8	100000 ≤	400	38.2
Married	221	21.1	**Occupation**		
Divorced	6	0.6	Full time Employed	248	23.7
Widow	5	0.5	Part time Employed	67	6.4
**Education**			Unemployed	102	9.7
No formal education	3	0.3	Home maker	59	5.6
Primary	5	0.5	Full time student	523	50.0
Secondary	46	4.4	Part time student	29	2.8
Higher secondary	125	11.9	Other	11	1.1
Undergraduate	665	63.5	**Region**		
Post graduate	203	19.4	Punjab	235	22.5
**Resident**			Sindh	562	53.7
Rural	97	9.3	KPK	135	12.9
Urban	871	83.2	Balochistan	1	0.1
Semi Urban	79	7.5	Islamabad	109	10.4
			Azad Jammu Kashmir	3	0.3
			Gilgit Baltistan	2	0.2

Gender was found to be significantly associated with depression (*p<0.01*) and anxiety *(p=.001)*. Education status was only significantly associated with depression (*p<0.001*). Place of residence and occupation were significantly associated with both depression (*p<0.001*) and anxiety (*p< 0.001*). Household income was significantly associated only with symptoms of depression (*p<0.01*). Previous psychiatric illness and previous psychiatric medications were significantly associated with depression (*p<0.001*) and anxiety (*p<0.001*) (Table 2).

**Table 2:** Association of depression and anxiety with sociodemographic characteristics

Variables	Depression			Anxiety		
	No *f(%)*	Yes *f(%)*	□^2^	No *f(%)*	Yes *f(%)*	□^2^
**Age (Years)**						
18 - 30	542(60.9)	348(39.1)	1.674	384(43.1)	506(56.9)	1.694
> 30	87 (55.4)	70 (44.6)		59 (37.6)	98 (62.4)	
**Gender**						
Male	320(64.4)	177(35.6)	7.327**	266(53.5)	231(46.5)	48.706***
Female	309(56.2)	241(43.8)		177(32.2)	373(67.8)	
**Marital Status**						
Single	498(61.1)	317(38.9)	5.925	350(42.9)	465(57.1)	5.596
Married	128(57.9)	93(42.1)		92(41.6)	129(58.4)	
Divorced	02 (33.3)	04 (66.7)		0 (0.0)	06(100.0)	
Widow	01 (20.0)	04 (80.0)		01 (20.0)	04 (80.0)	
**Education**						
No formal education	01 (33.3)	02 (66.7)	23.468**	02 (66.7)	01 (33.3)	9.324
Primary	01 (20.0)	04 (80.0)		01 (20.0)	04 (80.0)	
Secondary	27 (58.7)	19 (41.3)		20 (43.5)	26 (56.5)	
Higher secondary	57 (45.6)	68 (54.4)		40 (32.0)	85 (68.0)	
Undergraduate	401(60.3)	264(39.7)		284(42.7)	381(57.3)	
Post graduate	142(70.0)	61 (30.0)		96 (47.3)	107(52.7)	
**Resident**						
Rural	68 (70.1)	29 (29.9)	22.416**	59 (60.8)	38 (39.2)	18.571**
Urban	532(61.1)	339(38.9)		360(41.3)	511(58.7)	
Semi Urban	29 (36.7)	50 (63.3)		24 (30.4)	55 (69.6)	
**Occupation**						
Full time Employed	163(65.7)	85 (34.3)	14.252*	121(44.8)	127(51.2)	22.325*
Part time Employed	36 (53.7)	31 (46.3)		25 (37.3)	42 (62.7)	
Unemployed	47 (46.1)	55 (53.9)		31 (30.4)	71 (69.6)	
Home maker	34 (57.6)	25 (42.4)		13 (22.0)	46 (78.0)	
Full time student	323(61.8)	200(38.2)		230(44.0)	293(56.0)	
Part time student	16 (55.2)	13 (44.8)		13 (44.8)	16 (55.2)	
Other	10 (52.6)	09 (47.4)		10 (52.6)	09 (47.4)	
**Household monthly income**						
500000 ≥	180(52.6)	162(47.4)	12.401*	147(43.0)	195(57.0)	0.483
500001-100000	189(62.0)	116(38.0)		124(40.7)	181(59.3)	
100000 ≤	260(65.0)	140(35.0)		172(43.0)	228(57.0)	
**Smoker**						
Non smoker	559(60.9)	359(39.1)	2.074	392(42.7)	526(57.3)	0.526
Current smoker	50(54.3)	42(45.7)		37 (40.2	55 (5.98)	
Formal smoker	20 (54.1)	17 (45.9)		14 (37.8)	23 (62.2)	
**Psychiatric History**						
No	588(62.5)	353(37.5)	22.513**	432(45.9)	509(54.1)	49.275**
Yes	41 (38.7)	65 (61.3)		11 (10.4)	95 (89.6)	
**History of Medicine**						
No	617(61.7)	383(38.3)	24.483**	437(43.7)	563(56.3)	17.599**
Yes	12 (25.5)	35 (74.5)		06 (12.8)	41 (87.2)	

Binary logistic regressions were performed to determine any predictive association of socio-demographics with depression and/or anxiety. The analysis indicated that gender, education, residence, household income, previous psychiatric problems and previous psychiatric medication are the key factors associated with a significant increase in depression among the participants with odds ratios of 1.410 [1.099-1.809], 9.311 [1.020-85.030], 0.370 [0.229-0.596], 0.579 [0.227-1.480], 1.671 [1.244-2.246], 2.641 [1.748-3.989] and 4.711 [2.416-9.187], respectively. In addition, gender, place of residence, previous psychiatric problem, and previous psychiatric medications were found to be the key factors associated with a significant increase in depression with an odds ratio of 2.427 [1.888-3.119], 0.619 [0.376-1.019], 1.166 [0.458-2.969], 7.330 [3.876-13.863] and 5.313 [2.236-12.629], respectively (Table 3 and 4).

**Table 3:** Logistic regression analysis of the variables with depression

*Measures*	*OR*	*95% (CI)*	*p*
**Gender**			
Female ^Ref: Male^	1.410	1.099-1.809	0.007
**Education**			
No formal Edu. ^Ref: post graduate^	4.656	0.414-52.313	0.001
Primary ^Ref: post graduate^	9.311	1.020-85.030	
Secondary ^Ref: post graduate^	1.638	0.847-3.167	
Higher secondary ^Ref: post graduate^	2.777	1.749-4.410	
Undergraduate ^Ref: post graduate^	1.533	1.093-2.149	
**Resident**			
Rural ^Ref: Semi Urban^	0.247	0.132-0.465	0.001
Urban ^Ref: Semi Urban^	0.370	0.229-0.596	
**Occupation**			
Full time Employed ^Ref: others^	0.579	0.227-1.480	0.039
Part time Employed ^Ref: others^	0.957	0.345-2.655	
Unemployed ^Ref: others^	1.300	0.487-3.468	
Home maker ^Ref: others^	0.817	0.289-2.307	
Full time student ^Ref: others^	0.688	0.275-1.722	
Part time student ^Ref: others^	0.903	0.283-2.881	
**Household monthly income**			
500000 ≥ ^Ref: 100000<^	1.671	1.244-2.246	0.002
500001-100000 ^Ref: 100000<^	1.440	0.837-1.553	
**Previous Psychiatric Problem**			
Yes ^Ref: No^	2.641	1.748-3.989	0.001
**Previously Psychiatric Medication used**			
Yes ^Ref: No^	4.711	2.416-9.187	0.001

OR= odd ratio; CI= confidence interval; Ref= reference group

**Table 4:** Logistic regression analysis of the variables with anxiety

Measures	*OR*	*95% CI*	*p*
**Gender**			
Female ^Ref: Male^	2.427	1.888-3.119	0.001
**Resident**			
Rural ^Ref: Semi Urban^	0.281	0.150-0.527	0.001
Urban ^Ref: Semi Urban^	0.619	0.376-1.019	
**Occupation**			
Full time Employed ^Ref: others^	1.166	0.458-2.969	0.002
Part time Employed ^Ref: others^	1.867	0.668-5.216	
Unemployed ^Ref: others^	2.545	0.941-6.879	
Home maker ^Ref: others^	3.932	1.321-11.704	
Full time student ^Ref: others^	1.415	0.566-3.541	
Part time student ^Ref: others^	1.368	0.429-4.364	
**Previous Psychiatric Problem**			
Yes ^Ref: No^	7.330	3.876-13.863	0.001
**Previously Psychiatric Medication used**			
Yes ^Ref: No^	5.313	2.236-12.629	0.001

Out of the total sample population, 39.9% suffered from depression and 57.7% from anxiety (Table 5).

**Table 5:** Frequency distribution of Depression and Anxiety symptoms

Categories	Depression	Anxiety
No	629 (60.1)	443 (42.3)
Mild	262 (25.0)	266 (25.4)
Moderate	128 (12.2)	253 (24.2)
Severe	28 (2.7)	85 (8.1)

## Discussion

The present study indicated the significant prevalence of anxiety and depression in a sample of the general population of Pakistan during the COVID 19 outbreak from 5 May to 25 July 2020. Our study findings suggest that being a woman with a lower level of education, living in an urban area, occupation, previous psychiatric illness, and medication were significantly associated with symptoms of anxiety and depression. Our study findings suggest that women were more likely to be anxious and depressed (67.8% & 43.8%, respectively) than males (46.5% and 35.6%, respectively) during the lockdown. This result is supported by a study conducted by (Farooq et al., 2019) in which females were 2.5 times as anxious and depressed as males (39.4% vs. 23.3%, respectively). Another research (Zahidie & Jamali, 2013) found that the prevalence of anxiety and depressive symptoms were 29% and 66% among women, compared to 10% and 33% among men. These findings are backed by studies conducted globally which report higher anxiety symptoms among females in China (Zhou et al., 2020; Hou et al., 2020), India (Varshney et al., 2020), Oman (Badahdah et al., 2020) and Spain (González-Sanguino et al., 2020). Plausible reasons for the higher prevalence of anxiety and depression among women could be biological factors, socioeconomic disadvantage, loss of social status, maladapted coping strategies, and the lack of a support system for women in this country (Mirza & Jenkins, 2004). Other well-known reasoning may be that most women have to balance their household work and professional workload due to the inherited socio-cultural norms that still prevail in Pakistani households. Males are barely involved in household activities. As men spend more time at home due to the ‘stay home, stay safe’ policy of the government, the workload burden of the women in the household increases.

Moreover, anxiety and depression can be seen as more prevalent in urban and semi-urban locations. This could be because COVID-19 is more prevalent in urban settlements. Lockdown has had a great impact on all the densely populated cities of Pakistan, putting all the lives of the people living there on hold. Anxiety and depression can be significantly associated with the employment status of the general public, a local reflection of the hundreds of thousands of jobs being lost across the world. Pakistan’s Ministry of Finance revealed 3 million jobs had been lost during the COVD19 outbreak (Gulf news, 2020). The findings are supported by a Chinese study which showed that the prevalence of psychological health problems are more common among urban residents due to a great number of COVID-19 cases among cities and urban areas acting as epicenters of the diseases (Liu et al., 2021). Salary cuts and reductions in new jobs are expected. Moreover, uncertainty and possibly fear could have led to the development of more depression and anxiety symptoms. In addition, offices have been shut down because of the travel ban, and most employees are working from home. Lack of contact with co-workers could affect workers' motivation, satisfaction with work, and productivity. Not being able to meet deadlines and targets due to only earning hand of house and the usual pressure may cause a rise in anxiety levels among them.

Furthermore, household income is significantly associated with depression in the participants. The lower the household income, the more indicators of depressive symptoms there were. Sareen et al. (2011) found that low levels of household income are associated with mental disorders and suicide attempts, and a reduction in household income is associated with an increased risk of mental disorder incidents. One possible explanation for this could be that the lockdown imposed has disrupted the economic flow throughout the country.

Our results suggest that there is an increased prevalence of depression and anxiety among people with previous mental health problems and/or who were on medication for psychiatric disorders (p<0.001). One of the reasons for this is that the widespread lockdown has meant psychiatric patients are unable to contact their doctors in times of need. The closure of all psychiatric OPDs (Dawn news, 2020) and the current chaotic situation due to COVID-19 has increased the severity of some patients’ psychiatric conditions, leading to a spike in depression and anxiety among them. Also, being unable to travel and get medications during the lockdown is a reason for the spike.

Monitoring the mental health of populations during a pandemic is crucial, as public fear and fear induced by over-reacting behavior could act as a barrier to the control of infectious diseases (Dong & Bouey, 2020). In addition, the existing stringent lockdown measures and the uncertain period of home isolation reflect an ongoing traumatic occurrence that could potentially contribute to substantial long-term health costs. Therefore, epidemiological monitoring and targeted intervention should be introduced in good time to avoid more mental health issues in the future.

### Limitations of the Study

Along with the strong evidence this study has highlighted, there are some limitations and bias which are common with any cross-sectional study. Firstly, the researchers did not go into the field to collect data but used electronic means during the lockdown since the public health regulations were in place. Since the participation was voluntary, only the young adults who were active on social media had more participation resulting in lack of representation of all socio-demographic features. Therefore, the results cannot be generalized to the entire population of Pakistan. Secondly, most of the study sample was from 18 to 30 years of age leaving the gaps for the middle aged and older age groups. Thirdly, considering the high illiteracy rate of Pakistan, our sample size was mostly made up of a literal population. All of these factors mean the results are not generalizable as representative of the entire population. They do, however, offer important indicators, likely to be shared more generally,

## Conclusion

The present study reported the high prevalence of depression and anxiety in participants from a broad spectrum of the general population of Pakistan during the country-wide lockdown due to the COVID-19 pandemic. Mental Health Ordinance 2001 in Pakistan preserves the rights of citizens dealing with mental health issues and guarantees to take care of them. In these difficult times, the government of Pakistan should make mental health care one of its top priorities. Pakistan's government is doing its best to reduce the spread of the pandemic in the country; however, effective steps should also be taken for the care of mental health of its citizens.

## Data Availability

The data set is available upon request from the corresponding author.
